# Interventions to Decrease Carotid-Intima Media Thickness in Children and Adolescents With Type 1 Diabetes: A Systematic Review and Meta-Analysis

**DOI:** 10.3389/fcdhc.2022.882504

**Published:** 2022-07-04

**Authors:** Adina Mihaela Epure, Daniela Anker, Stefano Di Bernardo, Bruno R. da Costa, Nicole Sekarski, Arnaud Chiolero

**Affiliations:** ^1^ Population Health Laboratory (#PopHealthLab), University of Fribourg, Fribourg, Switzerland; ^2^ Department of Epidemiology and Health Services, Center for Primary Care and Public Health (UNISANTÉ), University of Lausanne, Lausanne, Switzerland; ^3^ Paediatric Cardiology Unit, Woman-Mother-Child Department, Lausanne University Hospital (CHUV), Lausanne, Switzerland; ^4^ Institute of Primary Health Care (BIHAM), University of Bern, Bern, Switzerland; ^5^ Institute of Health Policy, Management and Evaluation, University of Toronto, Toronto, Canada; ^6^ School of Population and Global Health, McGill University, Montréal, Canada

**Keywords:** diabetes, atherosclerosis, carotid intima-media thickness, children, trials

## Abstract

**Introduction:**

Hyperglycemia is associated with a higher cardiovascular risk, as evidenced by increased carotid-intima media thickness (CIMT) in youth with diabetes. We conducted a systematic review and meta-analysis to assess the effect of pharmacological or non-pharmacological interventions on CIMT in children and adolescents with prediabetes or diabetes.

**Methods:**

We conducted systematic searches of MEDLINE, EMBASE, and CENTRAL, together with supplementary searches in trial registers and other sources for studies completed up to September 2019. Interventional studies assessing ultrasound CIMT in children and adolescents with prediabetes or diabetes were considered for inclusion. Where appropriate, data were pooled across studies using random-effect meta-analysis. Quality was assessed using The Cochrane Collaboration’s risk-of-bias tool and a CIMT reliability tool.

**Results:**

Six studies involving 644 children with type 1 diabetes mellitus were included. No study involved children with prediabetes or type 2 diabetes. Three randomized controlled trials (RCTs) evaluated the effects of metformin, quinapril, and atorvastatin. Three non-randomized studies, with a before-and-after design, evaluated the effects of physical exercise and continuous subcutaneous insulin infusion (CSII). The mean CIMT at baseline ranged from 0.40 to 0.51 mm. The pooled difference in CIMT was -0.01 mm (95% CI: -0.04 to 0.01) for metformin compared to placebo (2 studies; 135 participants; I^2^: 0%). The difference in CIMT was -0.01 mm (95% CI: -0.03 to 0.01) for quinapril compared to placebo (1 study; 406 participants). The mean change from baseline in CIMT was -0.03 mm (95% CI: -0.14 to 0.08) after physical exercise (1 study; 7 participants). Inconsistent results were reported for CSII or for atorvastatin. CIMT measurement was rated at a higher quality on all reliability domains in 3 (50%) studies. The confidence in results is limited by the low number of RCTs and their small sample sizes, as well as the high risk of bias in before-and-after studies.

**Conclusions:**

Some pharmacological interventions may decrease CIMT in children with type 1 diabetes. However, there is great uncertainty with respect to their effects and no strong conclusions can be drawn. Further evidence from larger RCTs is required.

**Systematic Review Registration:**

PROSPERO, CRD42017075169

## 1 Introduction

Children and adolescents with diabetes have a high long-term cardiovascular risk (1, 2), as evidenced by signs of subclinical atherosclerosis and increased carotid-intima media thickness (CIMT) (3–6). Prevalence of both type 1 and type 2 diabetes has increased in the past decades (2, 7). This is particularly worrisome because diabetes clusters with several other risk factors, for instance, hypertension, hyperlipidemia, or microalbuminuria (8), which may track into adulthood (9, 10) and accelerate the process of atherosclerosis (11, 12). Also, increased CIMT in adulthood is associated with cardiovascular disease (CVD) events, such as heart attack and stroke (8, 13, 14). Early intervention for CVD prevention in children with diabetes is therefore paramount, yet complex and relatively understudied.

Clinical trials evaluating cardiovascular treatment efficacy in early life use surrogate markers of CVD, such as ultrasound CIMT. Several studies in adults showed that drug treatments or dietary interventions may slow progression of CIMT (15–17), which in turn may be associated with a reduction in CVD risk (15). Likewise, clinical trials in high-risk children with obesity or familial hyperlipidemia showed that exercise training (18) or statin therapy (18) may decrease CIMT. However, in children with diabetes, data on effective interventions are limited and clinical recommendations are largely based on expert opinions (19–21). A few clinical trials using CIMT were performed recently, but they were not part of a systematic review and meta-analysis. Additionally, CIMT measurement methods are heterogeneous at young ages and inconsistent findings across studies may be partly explained by a low measurement reliability (22).

We therefore conducted a systematic review and meta-analysis (1) to assess the effect of pharmacological or non-pharmacological interventions on CIMT in children and adolescents with prediabetes or diabetes and (2) to assess the characteristics and reliability of CIMT measurement methods used in the included studies.

## 2 Methods

### 2.1 Protocol Development and Reporting

This study is part of a larger systematic review project that focuses on prenatal and postnatal exposures or interventions and CIMT in children and adolescents (22, 23). We followed methods outlined in the research protocol for this systematic review project, which was also registered with the International Prospective Register of Systematic Reviews (PROSPERO) (registration number CRD42017075169) and published (23). The reporting of this paper complies with the Preferred Reporting Items for Systematic Reviews and Meta-Analyses (PRISMA) guidelines (24).

### 2.2 Eligibility Criteria

#### 2.2.1 Study Designs

Interventional studies with a randomized or non-randomized, controlled or non-controlled design were considered for inclusion.

#### 2.2.2 Participants

We considered for inclusion studies in children with a mean age ≤18 years at study entry and either prediabetes (e.g., impaired glucose tolerance, impaired fasting glucose) or diabetes mellitus (e.g., type 1 diabetes, type 2 diabetes, diabetes secondary to diseases of the exocrine pancreas, endocrinopathies, or drug-induced diabetes mellitus).

#### 2.2.3 Interventions

No restrictions were posed. Both pharmacological and non-pharmacological interventions were deemed equally eligible.

#### 2.2.4 Comparators

No restrictions were posed. Where applicable, the comparator could be a pharmacological or non-pharmacological intervention, usual care, placebo, or no intervention.

#### 2.2.5 Outcome Measures

The outcome was the intima-media thickness of the carotid artery measured by ultrasonography.

#### 2.2.6 Time Frame and Setting

No restrictions were posed.

#### 2.2.7 Language

Studies in English and French were considered for inclusion.

### 2.3 Search Strategy

Systematic searches were conducted in the Medical Literature Analysis and Retrieval System Online (MEDLINE) database, Excerpta Medica database (EMBASE), and Cochrane Central Register of Controlled Trials (CENTRAL) from inception to March 2019. Supplementary searches were performed in September 2019 and consisted of (1) a manual search of reference lists and other reviews on the topic, (2) forward citation tracking on Web of Science based on retrieved eligible reports, and (3) personalized search queries in Google Scholar and trial registers. The strategies for the systematic searches are provided in the published study protocol (23) and those for the supplementary searches in **Table S1** in **Supplementary Material**.

### 2.4 Study Selection Process

Study references were managed with Endnote (version X8.1) and deduplicated according to the method of Bramer et al. (25). Study screening was performed initially based on titles and abstracts and then based on full texts retained in the first step. Each report was screened independently by 2 reviewers using Covidence (26). Disagreements were resolved by discussion or, if necessary, by a third reviewer. The investigators of completed studies identified through supplementary searches in trial registers were contacted by email, but no supplementary data could be provided for this systematic review (**Table S2** in **Supplementary Material**).

### 2.5 Data Extraction

Data were extracted independently by 2 reviewers using an electronic form in Microsoft Excel (version 2016). Extracted information concerned (1) study and population characteristics, (2) CIMT measurement method and reliability, (3) intervention characteristics, (4) adjusted and unadjusted effect sizes, (5) methodological quality (or risk of bias). The methodological quality was evaluated using the Cochrane’s collaboration risk-of-bias tool for randomized studies (22, 27). This tool classifies studies at low, high, and unclear risk of bias for study design and conduction, which made us conclude on high, low, and unclear methodological quality, respectively (28). The quality of the CIMT measurement method was evaluated using the tool published in the study protocol (23), which evaluates (1) the site of measurement, (2) the image analysis methods, and (3) the assessment of measurement reproducibility. This tool classifies measurements at higher, lower, and unclear reliability, which made us conclude on higher, lower, and unclear quality, respectively. Disagreements between reviewers were resolved by discussion or with the arbitration of a third reviewer. Essential missing information was searched by checking additional references related to that study, such as the published research protocol. The corresponding author of one study was contacted by e-mail and provided complementary information for computing the effect size estimate (29). The certainty of the evidence was rated for each intervention type by 1 reviewer using GRADE (Grading of Recommendations, Assessment, Development and Evaluations) (30). The GRADE rating (high, moderate, low, very low certainty) specifies the extent to which one can be confident that an estimate of effect is close to the true effect and involves consideration of limitations within and across studies with regard to methodological quality, inconsistencies and imprecision in effects, indirectness of the evidence, or publication bias.

### 2.6 Data Analysis

Data analysis was performed in Stata (version 16), with graphical output from Stata (version 16) or R studio (version 4.1.2). Analyses were performed for each intervention type according to the study protocol (23) and recommendations and formulae provided in the Cochrane Handbook for Systematic Reviews (28, 31), Lipsey and Wilson (32), Wan and colleagues (33), Fu and colleagues (34), Sullivan (35), and Reichenbach and colleagues (36). Descriptive statistics about study participants are presented as means and standard deviations. For controlled studies, effect sizes are presented as differences in mean scores at follow-up or differences in mean change scores from baseline to follow-up with 95% confidence intervals (CI). For non-controlled studies, effect sizes are presented as mean change scores from baseline to follow-up with 95% CI. When necessary, data transformations were done: (1) means and standard deviations were estimated from medians and interquartile ranges (33); (2) the mean change score from baseline to follow-up for a single arm was calculated as *mean score at follow-up – mean score at baseline*; differences between arms were calculated as *intervention – comparison*; and (3) CIs were calculated from standard errors or estimated from p-values (28, 32, 35, 36). To perform the meta-analysis, additional data simplifications were done: (1) if a study reported on both mean and maximum CIMT, mean CIMT was included in the analysis; (2) if a study reported on both systolic and diastolic CIMT, the diastolic CIMT was included in the analysis; and (3) if a study provided effect estimates with different levels of adjustment, most adjusted estimates were used in the analyses. To report hemoglobin A1c (HbA1c) values as percentage (%) and mmol/mol, conversions were performed according to the National Glycohemoglobin Standardization Program’s (NGSP) converters (37) and underlying equations (38):

(1)*H/tiffb/tiffA*1*c*_*m/tiffm/tiffo/tiffl*/*m/tiffo/tiffl*_=(*H/tiffb/tiffA*1*c*_*%*_×10.929)−23.5
(2) $HbA1c_{\%}=(HbA1c_{mmol/mol}\times 0.09148)+2.152$

The meta-analysis was performed using the DerSimonian–Laird random-effect model, with the difference in CIMT in mm between intervention and comparison arms as the intervention effect. Pooling was not feasible for all studies because of the different intervention types assessed across studies. As previously shown to be valid, we pooled together outcomes reported as mean score at follow-up and mean change score from baseline to follow-up in the same meta-analysis (31, 39). The heterogeneity was assessed by the Cochran’s Q test, I^2^, and tau^2^ statistics (28, 40, 41). We planned to assess publication bias using funnel plots and Egger’s test (23), but this was not feasible due to the small number of included studies. We interpreted the point estimate as the best average treatment effect and reported alongside it the 95% confidence interval, which provides the uncertainty around the point estimate, as per the PRISMA guidelines (24) and recommendations of the Cochrane Collaboration (28, 31).

## 3 Results

### 3.1 Description of Studies and Baseline Characteristics of Participants

**Figure 1:**
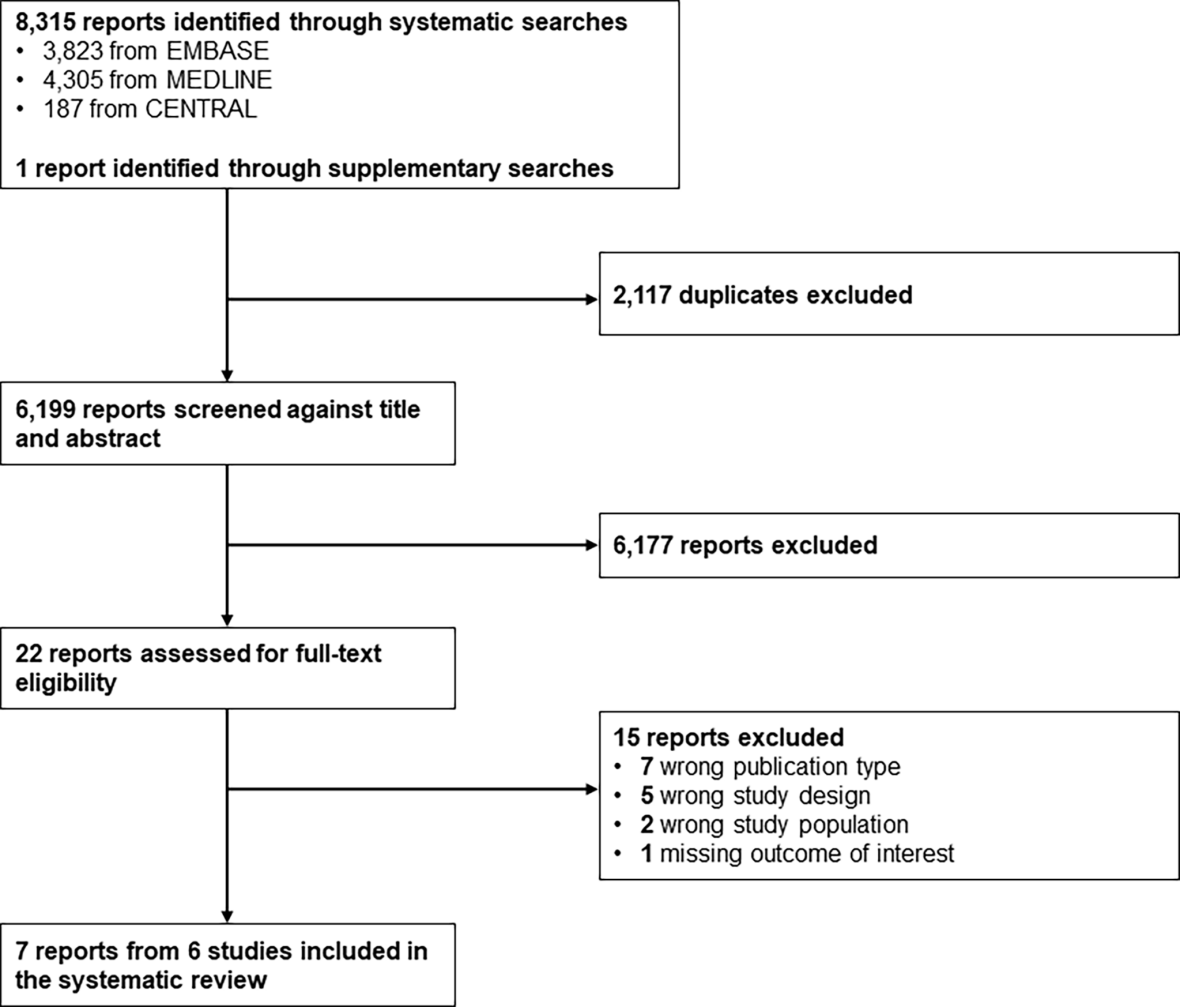
Study selection flow. CENTRAL, Cochrane Central Register of Controlled Trials; EMBASE, Excerpta Medica database; MEDLINE, Medical Literature Analysis and Retrieval System Online.

A total of 6,199 reports were screened based on titles and abstracts, and 22 were selected for full-text reviewing (**Figure 1**). Seven full texts, pertaining to 6 studies, with a randomized controlled design (n = 3) or a non-randomized non-controlled design (n = 3), were included in the systematic review. Studies were conducted in healthcare facilities in Europe (n = 2), Australia (n = 2), North America (n = 1), or cross-continentally (n = 1) (**Table 1**). Some 644 boys and girls (mean age between 10.9 and 17.3 years) with type 1 diabetes mellitus (mean time since diagnosis between 2.7 and 8.0 years) were included across studies. Mean HbA1c at baseline ranged from 63 to 74.67 mmol/mol (7.92 to 8.98%) (**Table 1**). Mean CIMT at baseline ranged from 0.40 to 0.51 mm. No study included children with prediabetes or type 2 diabetes (**Table S3** in **Supplementary Material**).

**Table 1:** Study and patients’ characteristics at baseline.

Author, year	Country	Design	Setting (N sites)	Intervention arms	N participants	Age, years	Male, %	BMI, kg/m^2^	Diabetes duration, years	HbA1c, mmol/mol	HbA1c, %
Anderson et al., 2017 (42)	Australia	Randomized controlled trial (parallel assignment)	Healthcare facility (multiple)	Metformin	45	14.00 (2.50)	46.67	N/S *^a^*	5.20 (3.60)	71.00 (16.08)	8.65 (1.47)
				Placebo	45	13.30 (2.60)	44.44	N/S *^a^*	5.80 (4.10)	74.67 (14.55)	8.98 (1.33)
Bjornstad et al., 2018 (43)	United States of America	Randomized controlled trial (parallel assignment)	Healthcare facility (multiple)	Metformin	25	17.30 (2.30)	44.00	25.40 (4.40)	8.00 (3.70)	72.00 (10.50)	8.74 (0.96)
				Placebo	23	15.90 (2.70)	56.52	25.30 (4.90)	7.80 (4.40)	69.00 (8.30)	8.46 (0.76)
Tolwinska et al., 2013 (44)	Poland	Before and after study (single arm)	Healthcare facility (multiple)	CSII	32	14.90 (2.50)	37.50	21.60 (2.80)	3.70 (3.20)	67.21 (18.58)	8.30 (1.70)
Harrington et al., 2013 (29)	Australia	Before and after study (single arm)	Healthcare facility (single)	CSII	22	12.50 (2.90)	40.91	N/S	3.40 (3.00)	70.49 (16.39)	8.60 (1.50)
Marcovecchio et al., 2017 (45)	Australia, Canada, United Kingdom	Randomized controlled trial (factorial assignment)	Healthcare facility (multiple)	Quinapril	222	12.40 (1.40)	54.95	21.30 (3.52)	N/S	67.21 (13.11)	8.30 (1.20)
				Placebo	221	12.40 (1.40)	53.39	21.20 (3.67)	N/S	68.30 (13.11)	8.40 (1.20)
Marcovecchio et al. , 2017 (45)	Australia, Canada, United Kingdom	Randomized controlled trial (factorial assignment)	Healthcare facility (multiple)	Atorvastatin	223	12.40 (1.40)	54.71	21.30 (3.78)	N/S	67.21 (14.21)	8.30 (1.30)
				Placebo	220	12.40 (1.40)	53.64	21.20 (3.39)	N/S	67.21 (13.11)	8.30 (1.20)
Seeger et al., 2011 (46)	Netherlands	Before and after study (single arm)	Healthcare facility (single)	Physical exercise	9 *^b^*	10.90 (1.50)	44.44	17.00 (2.40)	2.70 (3.10)	63.00 (12.00)	7.92 (1.10)

ACEI, angiotensin-converting enzyme inhibitor; BMI, body mass index; CSII, continuous subcutaneous insulin infusion; HbA1c, glycated hemoglobin; N, number; N/S, not specified.

Data are mean (standard deviation).

a BMI: Anderson, 2017 provides BMI z-score: 0.90 (0.60) for metformin arm and 0.90 (0.50) for comparison arm.

b N participants: 9 for all baseline characteristics except for BMI computed using data from 7 participants that completed the study.

### 3.2 Description of CIMT Measurement Methods

Image acquisition and analysis were relatively uniform across studies (**Tables 2**, **S4** in **Supplementary Material**). The CIMT was primarily measured on the common carotid artery (CCA) far wall, with the distances between the intima and media interfaces assessed automatically over a specific length. A total of 1 to 2 CIMT outcomes were reported in each study. Two studies reported both mean and maximum wall thickness, and one study reported both diastolic and systolic CIMT. Measurements of the left or right or combined left and right carotid sides were analyzed, with one study reporting left CIMT and right CIMT as 2 separate outcomes. Three studies were judged to be at higher CIMT reliability on all domains of measurement quality.

**Table 2:** CIMT measurement characteristics and reliability.

Author, year	N outcomes	Image acquisition			Image analysis			Reliability		
		Side	Segment	Wall	Edge detection, analysis of the distance between interfaces	Cardiac cycle phase	Wall thickness	Acquisition site	Analysis	Reproducibility assessment
Anderson et al. , 2017 (42)	2	Left and right	CCA	Far	Automatic/semiautomatic, automatic over a specific length	End-diastole	Mean; maximum	High	High	High
Bjornstad et al., 2018 (43)	2	N/S	N/S	Far	N/S, automatic over a specific length	Diastole; systole	N/S	Unclear	Unclear	Unclear
Tolwinska et al., 2013 (44)	1	Left and right	CCA	Far	N/S, N/S	N/S	N/S	High	Unclear	Unclear
Harrington et al., 2013 (29)	2	Left and right	CCA	Far	Automatic/semiautomatic, automatic over a specific length	End-diastole	Mean; maximum	High	High	High
Marcovecchio et al., 2017 (45)	2	Left; right	CCA	Far	N/S, automatic over a specific length	End-diastole	N/S	High	High	High
Seeger, 2011 (46)	1	Left	CCA	Far	Automatic/semiautomatic, automatic over a specific length	N/S	N/S	High	High	Unclear

CCA, common carotid artery; CIMT, carotid intima-media thickness; N, number; N/S, not specified.

### 3.3 Effects of Interventions

**Figure 2:**
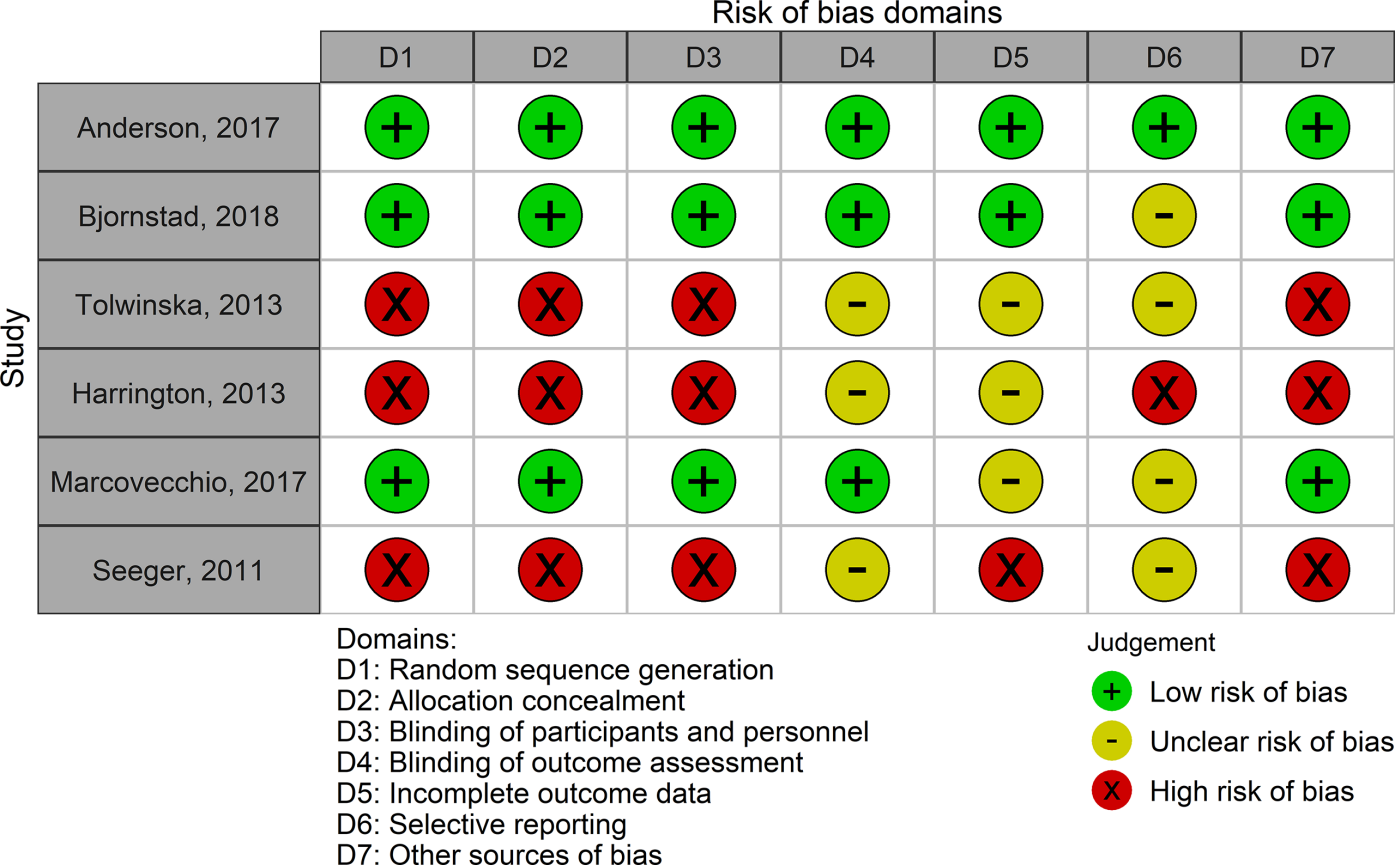
Risk of bias in each study included in the systematic review. Low risk of bias corresponds to high methodological quality. High risk of bias corresponds to low methodological quality.

The pharmacological interventions evaluated were metformin (antidiabetic drug), quinapril (angiotensin-converting enzyme inhibitor (ACEI) drug), atorvastatin (lipid-lowering drug), and continuous subcutaneous insulin infusion (CSII) (antidiabetic device). Physical exercise was the only non-pharmacological intervention evaluated (**Table 3**). Trials varied in their methodological quality, but the evidence from the 3 RCTs was generally at low risk of bias, with 1 or 2 domains at unclear risk of bias. One RCT was rated at low risk of bias on all domains (**Figures 2**, **S1** in supplementary material).

**Table 3:** The effect of interventions on CIMT in children with type 1 diabetes.

Author, year	Intervention type	CIMT definition	Pre-intervention CIMT, mm		Post-intervention effect, mm			
			Intervention mean (SD)	Placebo mean (SD)	N analyzed	Measure *^a^*	Estimate (95% CI)	Adjustments
Anderson et al., 2017 (42)	Metformin	Left and right CCA-segment far-wall (mean thickness, end-diastole)	0.40 (0.10)	0.40 (0.10)	90 (45 vs. 45)	Difference in mean scores at follow-up	-0.01 (-0.04 to 0.01)	Sex, age, HbA1c *^b^*
		Left and right CCA-segment far-wall (maximum thickness, end-diastole)	0.50 (0.10)	0.50 (0.10)	90 (45 vs. 45)	Difference in mean scores at follow-up	-0.01 (-0.04 to 0.02)	Sex, age, HbA1c *^b^*
Bjornstad et al., 2018 (43)	Metformin	N/S-side N/S-segment far-wall (N/S thickness, diastole)	0.47 (0.06)	0.46 (0.06)	45 (24 vs. 21)	Difference in mean change scores from baseline to follow-up	-0.03 (-0.08 to 0.02)	Baseline CIMT, change in body mass index, change in glucose infusion rate/insulin, change in systolic blood pressure
		N/S-side N/S-segment far-wall (N/S thickness, systole)	0.44 (0.06)	0.44 (0.06)	45 (24 vs. 21)	Difference in mean change scores from baseline to follow-up	0.00 (-0.05 to 0.05)	Baseline CIMT, change in body mass index, change in glucose infusion rate/insulin, change in systolic blood pressure
Tolwinska et al., 2013 (44)	CSII	Left and right CCA-segment far-wall (N/S thickness, N/S cardiac cycle phase)	0.51 (0.05)	N/A	32	Mean change score from baseline to follow-up	-0.02 (-0.03 to -0.01)	No adjustments
Harrington et al., 2013 (29)	CSII	Left and right CCA-segment far-wall (mean thickness, end-diastole)	0.41 (0.05)	N/A	22	Mean change score from baseline to follow-up	0.01 (-0.06 to 0.08)	No adjustments
		Left and right CCA-segment far-wall (maximum thickness, end-diastole)	N/S	N/A	22	Mean change score from baseline to follow-up	N/S *^c^*	No adjustments
Marcovecchio et al., 2017 (45)	Quinapril	Left CCA-segment far-wall (N/S thickness, end-diastole)	0.44 (0.05)	0.44 (0.06)	406 (204 vs. 202)	Difference in mean scores at follow-up	-0.01 (-0.03 to 0.01)	Child sex, age, duration of disease, log10 albumin/creatinine ratio, total cholesterol, HbA1c, country
		Right CCA-segment far-wall (N/S thickness, end-diastole)	0.44 (0.05)	0.44 (0.05)	406 (204 vs. 202)	Difference in mean scores at follow-up	-0.01 (-0.03 to 0.01)	Child sex, age, duration of disease, log10 albumin/creatinine ratio, total cholesterol, HbA1c, country
Marcovecchio et al., 2017 (45)	Atorvastatin	Left CCA-segment far-wall (N/S thickness, end-diastole)	0.44 (0.05)	0.44 (0.05)	406 (209 vs. 197)	Difference in mean scores at follow-up	-0.01 (-0.02 to 0.01)	Child sex, age, duration of disease, log10 albumin/creatinine ratio, total cholesterol, HbA1c, country
		Right CCA-segment far-wall (N/S thickness, end-diastole)	0.44 (0.05)	0.44 (0.05)	406 (209 vs. 197)	Difference in mean scores at follow-up	0.00 (-0.02 to 0.02)	Child sex, age, duration of disease, log10 albumin/creatinine ratio, total cholesterol, HbA1c, country
Seeger et al., 2011 (46)	Physical exercise	Left CCA-segment far-wall (N/S thickness, N/S cardiac cycle phase)	0.44 (0.09)	N/A	7	Mean change score from baseline to follow-up	-0.03 (-0.14 to 0.08)	No adjustments

ACEI, angiotensin-converting enzyme inhibitor; CIMT, carotid intima-media thickness; CSII, continuous subcutaneous insulin infusion; N, number; N/A, not applicable; N/S, not specified; SD, standard deviation.

a Change score from baseline to follow-up was calculated as value at follow-up – value at baseline; where applicable, differences between groups were calculated as intervention – placebo.

b Analyses were performed using linear mixed effect models including treatment group, time, and their interaction in the models.

c Insufficient data to compute an effect size; reported p-value = 0.82.

#### 3.3.1 Pharmacological Interventions

**Figure 3:**
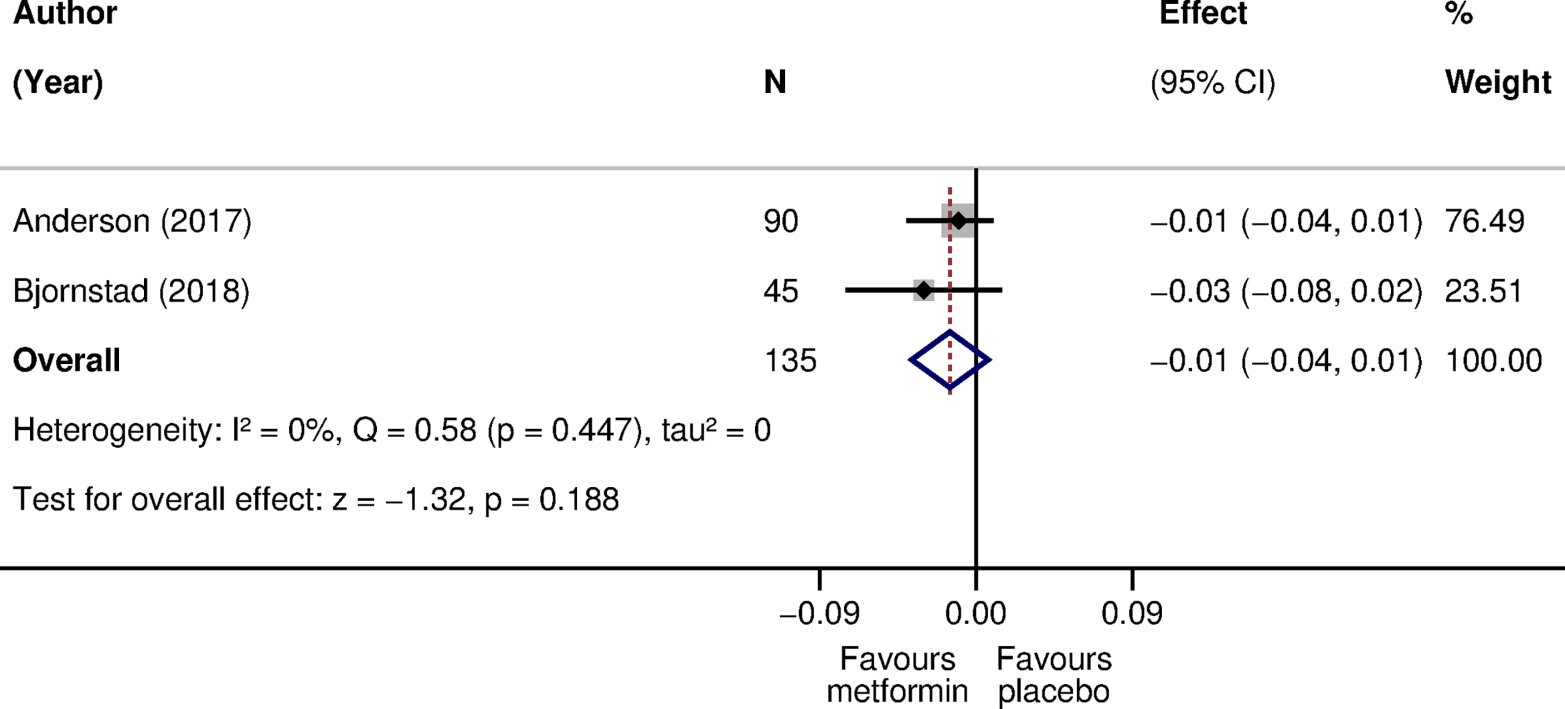
The effect of metformin compared with placebo on CIMT in children with type 1 diabetes. The effect is the difference in CIMT mean scores at follow-up (42) or in mean change scores from baseline to follow-up (43) in mm. A negative effect size corresponds to a lower CIMT in the metformin arm as opposed to the placebo arm. Weights are from the random-effects model. CI, confidence interval; CIMT, carotid intima-media thickness; N, sample size; Q, Cochran’s Q statistic; p, p-value; z, z statistic for the overall effect.

Two parallel-design RCTs compared metformin with placebo. The treatment duration ranged from 3 to 12 months (**Table S3** in **Supplementary Material**). The pooled difference in CIMT was -0.01 mm (95% CI: -0.04 to 0.01) in favor of metformin (135 participants; I^2^: 0%; tau^2^:0) (**Figure 3**).

One RCT with a 2-by-2 factorial design compared quinapril or atorvastatin with placebo in 406 children at high risk for diabetic nephropathy. Treatment was provided over 2 to 4 years (**Table S3**). The difference in CIMT was -0.01 mm (95% CI: -0.03 to 0.01) in favor of quinapril for either left or right CIMT. The difference in CIMT was -0.01 mm (95% CI: -0.02 to 0.01) in favor of atorvastatin for left CIMT, but not for right CIMT [0.00 mm (95% CI: -0.02 to 0.02)] (**Table 3**).

Two non-randomized non-controlled studies compared CIMT before and after initiation of treatment with CSII. The treatment duration was 6 months in one study and was not specified in the other study (**Table S3** in **Supplementary Material**). Effects in the opposite direction were reported. The mean change from baseline in CIMT was -0.02 mm (95% CI: -0.03 to -0.01) in one study (32 participants) and 0.01 mm (95% CI: -0.06 to 0.08) in the other study (22 participants) (**Table 3**).

#### 3.3.2 Non-Pharmacological Interventions

A single non-randomized non-controlled study compared CIMT before and after physical exercise for 18 weeks (**Table S3** in supplementary material). The mean change from baseline in CIMT was -0.03 mm (95% CI: -0.14 to 0.08) (7 participants) (**Table 3**).

### 3.4 Certainty of the Evidence

For metformin, quinapril, and atorvastatin, the evidence came from RCTs, but it was eventually rated at low certainty. For metformin, the evidence was downgraded due to very serious concerns related to imprecision in effect estimates (low number of participants; 95% CI of the pooled effect estimate crossing the line of no effect). For quinapril or atorvastatin, the evidence was downgraded due to serious concerns related to the comparator indirectness and imprecision in effect estimates. More specifically, quinapril and atorvastatin were evaluated in a 2-by-2 factorial design that assumed no interaction between the factorial comparisons. This means that quinapril was evaluated against a comparator comprising participants taking placebo and placebo or placebo and atorvastatin. Likewise, atorvastatin was evaluated against a comparator comprising participants taking placebo and placebo or placebo and quinapril. Regarding imprecision, the 95% CIs for the effect estimates indicate that no effect remains plausible despite the relatively large sample size (406 participants).

For CSII and physical exercise, the evidence came from non-randomized, non-controlled studies and it was rated at very low certainty. This rating was due to very serious concerns regarding the risk of bias (studies were rated at high or unclear risk of bias on all methodological domains), comparator indirectness (a single group of participants serving as their own controls), and imprecision. For CSII, the certainty of the evidence was also downgraded due to serious concerns related to inconsistency in results across studies.

## 4 Discussion

### 4.1 Summary of Main Results

In this systematic review of 6 interventional studies involving 644 children and adolescents with type 1 diabetes, we identified a small and statistically non-significant decrease in CIMT after metformin (low certainty), quinapril (low certainty), or physical exercise (very low certainty). Inconsistent results were reported for CSII or for atorvastatin. The CIMT measurement reliability was either higher or unclear. The confidence in results is limited by the low number of RCTs and their small sample sizes, as well as the high risk of bias in before and after studies.

### 4.2 Comparison With Other Studies

We found some evidence on the effect of medications in children with type 1 diabetes that was partially in line with findings among other children or adults at high CVD risk. Metformin was evaluated in a recent systematic review and meta-analysis that reported a pooled difference in CIMT of -0.053 mm (95% CI: -0.115 to 0.009) in favor of metformin among adults with prediabetes or diabetes (6 trials; 806 participants) (17). Our effect estimates in children with type 1 diabetes consistently pointed toward decreases in CIMT, but they were much smaller in magnitude and highly imprecise. The comparison of ACEI with placebo in a meta-analysis of 3 trials including 2,087 adults with impaired glucose tolerance, type 2 diabetes, or albuminuria showed no effect on CIMT (pooled difference 0.00 mm (95% CI: -0.01 to 0.00) (16). We found an effect estimate for ACEI in children with type 1 diabetes that was slightly higher in magnitude, but more imprecise (-0.01 mm; 95% CI: -0.03 to 0.01) (45). Likewise, the comparison of statins with placebo or usual care in a meta-analysis of 13 primary prevention trials showed a pooled difference in CIMT of 0.00 mm (95% CI: -0.01 to 0.01) in favor of statins (47). Nonetheless, one RCT among 211 children with familial hypercholesterolemia (48) showed that 2 years of pravastatin was associated with a -0.01mm (95% CI: -0.03 to 0.00) difference in CIMT. This latter trial was powered on CIMT, which was defined as the mean of the right and left CCA, carotid bulb, and internal carotid artery segments (49). Our results for the effect of atorvastatin on CIMT of the left (-0.01 mm; 95% CI: -0.02 to 0.01) and right (0.00 mm; 95% CI: -0.02 to 0.02) CCAs were inconsistent (45).

We found that the effect of dietary or lifestyle measures on CIMT is largely understudied in children with diabetes. We identified one non-controlled non-randomized study (7 participants) reporting a mean change in CIMT of -0.03 mm (95% CI: -0.14 to 0.08) following 18 weeks of exercise training. The study had several caveats, primarily related to the lack of an external control group and the extremely low sample size. Much stronger evidence exists in other populations. For instance, Garcia-Hermoso and colleagues (18) performed a systematic review and meta-analysis of 6 RCTs involving 303 children with overweight and obesity and reported that exercise training decreased CIMT by -0.31 standard deviation units (95% CI -0.54 to -0.07). Lifestyle interventions merit further consideration in future trials because they may be more acceptable to children and parents, may contribute to the development of healthy behaviors that track into adulthood (50), and have the potential to act on multiple mechanisms of atherosclerosis for instance, physical exercise may improve endothelial dysfunction and healthy diets may improve the lipid profile (51).

Adequately powered trials would be needed to identify suitable interventions to reduce CVD risk in children with type 1 diabetes. The RCTs included in our systematic review were not primarily designed to show an effect on CIMT, but on markers of endothelial dysfunction (flow-mediated dilation) (42), insulin sensitivity (steady-state glucose infusion rate/insulin) (43), or albuminuria (albumin-to-creatinine ratio) (45). However, they provide useful information to guide the design of future trials. Some of the interventions that were administered for at least 12 months, such as metformin or atorvastatin, resulted in point estimates for the treatment effect of about -0.01 mm. Although small, a decrease in CIMT of 0.01 mm/year might be clinically important on the long term as highlighted by the study of Willeit and colleagues (15). If we perform a rough estimation of the sample size required to have 80% power to detect a difference of 0.01 mm between 2 arms, at a 2-sided 0.05 α-level, when assuming a CIMT standard deviation of 0.05 mm, we would obtain a total of 788 participants (394 per arm). Increasing or decreasing the assumed value for the standard deviation of the outcome would result in a higher or lower sample size needed. The sources of variability for each study therefore need to be carefully considered in sample size planning (52).

### 4.3 Strengths and Limitations

To the best of our knowledge, this is the first quantitative synthesis of the effect of metformin on CIMT in children and adolescents with type 1 diabetes. Other strengths to be noted include the reporting of detailed characteristics of the CIMT measurement and broad searches, in multiple sources, to retrieve completed studies. However, the high imprecision in effects, together with the variation in trial designs and levels of methodological quality, limit the degree of confidence in results. Further, only children with type 1 diabetes were included in these studies. Our conclusions might not be applicable to patients with type 2 diabetes or prediabetes, although emerging evidence shows that the prevalence of increased CIMT is also high for adolescents and young adults with newly diagnosed type 2 diabetes (5). Next, measurement error in CIMT cannot be excluded and co-medications and co-interventions beyond the studied interventions were provided to participants, which may have influenced the observed effects (53). In fact, 3 out of 4 studies evaluating metformin, quinapril, and atorvastatin reported that insulin was continued during the study course and adjusted as per need or routinely recommended by the healthcare providers, which may have triggered imbalances between the intervention arms. One trial evaluating metformin for 12 months also reported providing dietary advice at baseline and 3 months, but this co-intervention was standardized and given to both the active and comparison arms. The risk of bias due to confounding is particularly important for non-randomized non-controlled studies due to the lack of a control group to account for time trends, no randomization and concealed allocation, and unadjusted effect estimates. However, the meta-analysis for metformin was performed using most adjusted estimates from 2 RCTs. Although the adjustment factors differed between the studies, there was no statistical heterogeneity associated with the pooled effect (I^2^: 0%; tau^2^: 0). Finally, the restriction to studies published in English or French, which were the languages spoken in common by the reviewers, is another limitation of this systematic review.

## 5 Conclusions

### 5.1 Implications for Practice and Research

Children with diabetes are at high risk of subclinical vascular complications, such as increased CIMT, and bear a disproportionate risk of clinical CVD in adulthood (12). This constitutes an important public health problem in the context of population aging and increased prevalence of diabetes worldwide (7), hence, early-life prevention of CVD has been advocated (2, 54). Multiple factors seem to contribute to their higher CVD risk, such as hyperglycemia, hypertension, dyslipidemia, or insulin resistance (55). Our meta-analysis suggests that the use of metformin as an adjunctive therapy may hold promise in CVD risk reduction in children with type 1 diabetes through improvements in CIMT. Provided the effect of metformin is confirmed in future trials, this is an important finding for clinical practice as vascular remodeling may constitute an additional treatment target. Given its insulin-sensitizing properties (43, 56), metformin may also have the ability to help with glycemic control during puberty when insulin resistance worsens and many youth with type 1 diabetes fail to meet clinical guidelines (57, 58).

Further evidence is needed to identify appropriate intervention strategies for maintaining a low CVD over the life course. The current evidence from RCTs reported on CIMT as a secondary endpoint and suffers from low certainty, mainly due to imprecision. The non-RCT evidence suffers from very low certainty, mainly due to high risk of bias and imprecision. Therefore, larger, adequately powered, and well-conducted RCTs, carried over longer time periods, in children with type 1 diabetes are warranted. Further evidence on the effect of nutrition, exercise, and psychosocial and behavioral interventions on preventing or improving vascular remodeling in youth with diabetes or prediabetes is also needed.

## Author Contributions

AE, NS, and AC designed the study, with input from BC and SB. AE carried out the literature searches. AE and DA performed the duplicate study selection, data extraction, and quality assessments. AC and NS resolved the screening and data extraction conflicts. AE carried out the statistical analyses, with input and supervision from BC and AC. AE wrote the first draft of the manuscript. AC, BC, DA, NS, and SB made critical revisions to the manuscript for important intellectual content. All authors contributed to the article and approved the submitted version.

## Funding

This study was funded by the Swiss National Science Foundation (www.snf.ch; project number 32003B-163240; grantee: AC). The funding body had no role in the study design, collection, analysis, and interpretation of data, or in the writing of the manuscript.

## Conflict of Interest

The authors declare that the research was conducted in the absence of any commercial or financial relationships that could be construed as a potential conflict of interest.

## Publisher’s Note

All claims expressed in this article are solely those of the authors and do not necessarily represent those of their affiliated organizations, or those of the publisher, the editors and the reviewers. Any product that may be evaluated in this article, or claim that may be made by its manufacturer, is not guaranteed or endorsed by the publisher.

## Abbreviations

ACEI, angiotensin-converting enzyme inhibitor; CCA, common carotid artery; CENTRAL, Cochrane Central Register of Controlled Trials; CI, confidence interval; CIMT, carotid-intima media thickness; CSII, continuous subcutaneous insulin infusion; CVD, cardiovascular disease; EMBASE, Excerpta Medica database; GRADE, Grading of Recommendations, Assessment, Development and Evaluations; HbA1c, Hemoglobin A1c; MEDLINE, Medical Literature Analysis and Retrieval System Online; NGSP, National Glycohemoglobin Standardization Program; PRISMA, Preferred Reporting Items for Systematic Reviews and Meta-Analyses; PROSPERO, International Prospective Register of Systematic Reviews; RCT, randomized controlled trial.
