# Supplementary material for: Interventions to Decrease Carotid-Intima Media Thickness in Children and Adolescents With Type 1 Diabetes: A Systematic Review and Meta-Analysis
Source: Front Clin Diabetes Healthc. 2022 Jul 4;3:882504. doi: 10.3389/fcdhc.2022.882504 (PMC10012108; doi:10.3389/fcdhc.2022.882504)
Supplement: Supplementary file 1 [file DataSheet_1.docx]

Supplementary Material

**1 Supplementary Data**

**1.1 Supplementary Tables**

**Table S1** Search strategies for supplementary searches.

| Source | Search Strategy |
| --- | --- |
|  |  |
| [www.clinicaltrials.gov](http://www.clinicaltrials.gov) | (baby OR newborn OR infant OR child OR adolescent) AND carotid AND (intima OR intimal) AND (media OR medial) AND (thickness OR thickening OR complex) AND (diabetes OR prediabetes OR dysglycemia OR "impaired glucose")  Additional filter: Study type=Interventional |
| [www.isrctn.com](http://www.isrctn.com) | (baby OR newborn OR infant OR child OR adolescent) AND carotid AND (intima OR intimal) AND (media OR medial) AND (thickness OR thickening OR complex) AND (diabetes OR prediabetes OR dysglycemia OR "impaired glucose") |
| [www.clinicaltrialsregister.eu](http://www.clinicaltrialsregister.eu) | (baby OR newborn OR infant OR child OR adolescent) AND carotid AND (intima OR intimal) AND (media OR medial) AND (thickness OR thickening OR complex) AND (diabetes OR prediabetes OR dysglycemia OR "impaired glucose") |
| Google Scholar  <https://scholar.google.com/> | infants\|children\|adolescents\|pediatric vascular\|cardiovascular\|atherosclerosis\|"intima\|intimal media\|medial thickness\|thickening\|complex" diabetes\|prediabetes\|"impaired glucose"\|dysglycemia trial\|intervention\|therapy\|treatment\|medication |
| Reference lists | Reference lists of all full-texts included after systematic searches (n=7) were searched for potentially eligible titles. |
| Web of Science | Forward citation tracking was performed based on all full-texts included after systematic searches (n=7). No date limits were applied. |

**Table S2** Completed studies retrieved via supplementary searches on trial registers.

| **Trial Registry name, study identifier** | **Study name (acronym)** |
| --- | --- |
| ClinicalTrials.gov,  NCT01597154 | Beating Diabetes Together: A Randomized Controlled Trial for Intensive Lifestyle Therapy for Youth with Type 2 Diabetes (BDT) |
| ClinicalTrials.gov, NCT02637154 | Motivational Interview in Adolescents with Poorly Controlled Type 1 Diabetes (MoHa) |

**Table S3** Additional study characteristics.

| Author, year | Target disease | | | Target intervention | | | Other treatments during the study course |
| --- | --- | --- | --- | --- | --- | --- | --- |
|  | Type | Diagnosis | Associated vascular complications | Type | Maximum dose & frequency | Duration |  |
| Anderson, 2017 [1] | type 1 diabetes | criteria and thresholds: N/S; timing of diagnosis: at least 6 months prior to study inclusion | N/S | Metformin | 2000 mg (weight ≥ 60 kg) or 1000 mg (weight < 60 kg) per day | 12 months | insulin (MDI or CSII) was continued and adjusted as per need; participants received dietary advice for 60 minutes from one dietitian at baseline (standardized information on healthy diet portion sizes, regular meals and physical activity) and at 3 months (standardized interactive nutrition session comparing relative nutritional value of healthy snack foods to 'sometimes' and 'treat' snack foods) |
| Bjornstad, 2018 [2] | type 1 diabetes | criteria and thresholds: ≥ 1 diabetes mellitus-associated autoantibody and insulin requirement since diagnosis (American Diabetes Association definition); timing of diagnosis: at least 12 months prior to study inclusion | N/S | Metformin | 2000 mg per day | 3 months | insulin was continued and adjusted as routinely recommended by the healthcare providers; participants were asked not to make changes to the type or insulin delivery mode (shots versus pump) and not to make any significant diet or activity changes during the intervention period |
| Tolwinska, 2013 [3] | type 1 diabetes | criteria and thresholds: N/S; timing of diagnosis: N/S | no | CSII | N/S | 6 months | N/S |
| Harrington, 2013 [4] | type 1 diabetes | criteria and thresholds: N/S; timing of diagnosis: at least 6 months prior to study inclusion | no | CSII | N/S | N/S | N/S |
| Marcovecchio, 2017 [5] | type 1 diabetes | criteria and thresholds: N/S; timing of diagnosis: at least 12 months prior to study inclusion or within the past year with an undetectable C-peptide level | high risk for diabetic nephropathy (adjusted albumin-to-creatinin ratio in the upper third of the screened population) | Quinapril | 10 mg per day | 2 to 4 years | insulin (MDI or CSII) was continued and adjusted as routinely recommended by healthcare providers; due to the factorial design of the trial, some participants also received a statin (10 mg per day) |
| Marcovecchio, 2017 [5] | type 1 diabetes | criteria and thresholds: N/S; timing of diagnosis: at least 12 months prior to study inclusion or within the past year with an undetectable C-peptide level | high risk for diabetic nephropathy (adjusted albumin-to-creatinin ratio in the upper third of the screened population) | Atorvastatin | 10 mg per day | 2 to 4 years | insulin (MDI or CSII) was continued and adjusted as routinely recommended by healthcare providers; due to the factorial design of the trial, some participants also received an ACE inhibitor (up to 10 mg per day) |
| Seeger, 2011 [6] | type 1 diabetes | criteria and thresholds: N/S; timing of diagnosis: N/S | N/S | Physical exercise | 2 sessions per week: 1 guided session (30 minutes of interval running and 30 minutes of group-based activities) and 1 individual session (30 minutes of interval running and a 10-minute warm-up and cooling down) | 18 weeks | N/S |
| Abbreviations: ACEI, angiotensin-converting-enzyme inhibitor; CSII, continuous subcutaneous insulin infusion; MDI, multiple daily injections; N/S, not specified. | | | | | | | |

**Table S4** CIMT equipment and operators.

|  | **US device** | **US mode** | **Transducer array, frequency (MHz)** | **Operators** |
| --- | --- | --- | --- | --- |
| Anderson, 2017 [1] | Phillips iU22 | B-mode | linear, 17 to 5 | Sonographers: experienced, trained, and led by one of the study authors (Roger Gent); they have been trained and undergone an accreditation process to evaluate the quality of their scans for this study in 2009. Readers: N/S. |
| Bjornstad, 2018 [2] | General Electric Vivid 7 | B-mode | N/S, N/S | Cardiologist with extensive experience in arterial imaging (Uyen Truong). |
| Tolwinska, 2013 [3] | Hewlett Packard SONOS 4500 | N/S | linear, 7.5 | A study author (Joanna Tolwinska). |
| Harrington, 2013 [4] | Philips iU22 | B-mode | linear, 17 | N/S |
| Marcovecchio, 2017 [5] | N/S | N/S | N/S, N/S | Sonographers: accredited before the study commenced; sonographer training was conducted through the Vascular Physiology Unit at University College London, which has extensive experience in running large-scale, vascular phenotyping trials in children and included a 1-week intensive training course in London for all study sonographers. Reader: centralized analysis by a single reader in a core laboratory (Vascular Physiology Unit at University College London). |
| Seeger, 2011 [6] | T3000 | B-mode | N/S, N/S | N/S |
| Abbreviations: CIMT, carotid intima-media thickness; N/S, not specified. | | | | |

## Supplementary Figures


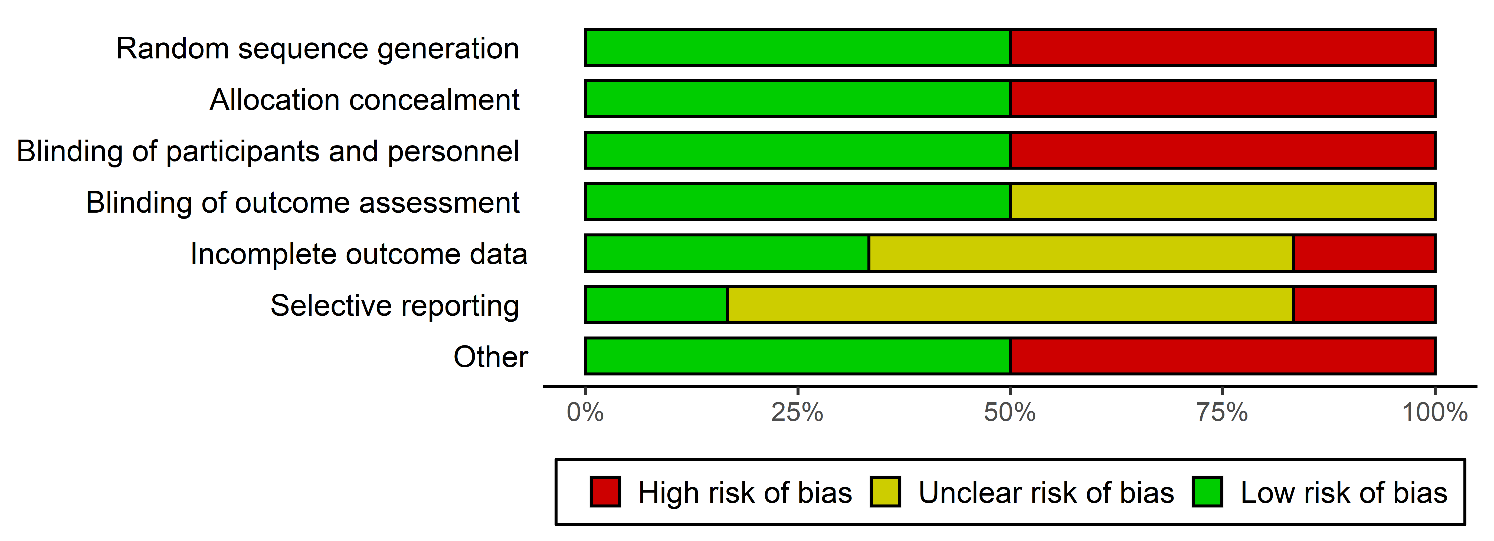


**Fig. S1.** Risk of bias across studies included in the systematic review. Low risk of bias corresponds to high methodological quality. High risk of bias corresponds to low methodological quality.

## References

1. Anderson JJA, Couper JJ, Giles LC, Leggett CE, Gent R, Coppin B, et al. Effect of Metformin on Vascular Function in Children With Type 1 Diabetes: A 12-Month Randomized Controlled Trial. The Journal of clinical endocrinology and metabolism. 2017;102(12):4448-56. Available from: <https://dx.doi.org/10.1210/jc.2017-00781>.

2. Bjornstad P, Schäfer M, Truong U, Cree-Green M, Pyle L, Baumgartner A, et al. Metformin Improves Insulin Sensitivity and Vascular Health in Youth With Type 1 Diabetes Mellitus. Circulation. 2018;138(25):2895-907. Available from: <https://dx.doi.org/10.1161/circulationaha.118.035525>.

3. Tołwińska J, Głowińska-Olszewska B, Bossowski A. Insulin therapy with personal insulin pumps and early angiopathy in children with type 1 diabetes mellitus. Mediators of inflammation. 2013;2013:791283. Available from: <https://dx.doi.org/10.1155/2013/791283>.

4. Harrington J, Peña AS, Wilson L, Gent R, Dowling K, Baghurst P, et al. Vascular function and glucose variability improve transiently following initiation of continuous subcutaneous insulin infusion in children with type 1 diabetes. Pediatr Diabetes. 2013;14(7):504-11. Available from: <https://dx.doi.org/10.1111/pedi.12050>.

5. Marcovecchio ML, Chiesa ST, Bond S, Daneman D, Dawson S, Donaghue KC, et al. ACE Inhibitors and Statins in Adolescents with Type 1 Diabetes. The New England journal of medicine. 2017;377(18):1733-45. Available from: <https://dx.doi.org/10.1056/NEJMoa1703518>.

6. Seeger JP, Thijssen DH, Noordam K, Cranen ME, Hopman MT, Nijhuis-van der Sanden MW. Exercise training improves physical fitness and vascular function in children with type 1 diabetes. Diabetes Obes Metab. 2011;13(4):382-4. Available from: <https://dx.doi.org/10.1111/j.1463-1326.2011.01361.x>.
